# Evaluation of Publication of Pediatric Drug Trials

**DOI:** 10.1001/jamanetworkopen.2021.5829

**Published:** 2021-04-14

**Authors:** Ananya Srivastava, Florence T. Bourgeois

**Affiliations:** 1 Faculty of Medicine, University of Toronto, Toronto, Ontario, Canada; 2 Pediatric Therapeutics and Regulatory Science Initiative, Computational Health Informatics Program, Boston Children’s Hospital, Boston, Massachusetts; 3 Department of Pediatrics, Harvard Medical School, Boston, Massachusetts

## Abstract

This cross-sectional study assesses the rate of publication of pediatric drug trials and examines the information lost when trials are not published.

## Introduction

Nonpublication of clinical trials compromises the integrity of scientific evidence and represents a breach in ethical obligations to trial participants.^1^ Timely publication of trials in the medical literature is especially important for pediatric trials, which are often challenging to conduct because of small participant pools and unique ethical and practical considerations. Pediatric trials frequently fill critical gaps in medical knowledge. Many medications used in pediatric populations, for example, have not been formally tested in children; therefore, essential data on pediatric safety and efficacy are not available.^2^ To increase our understanding of publication practices for pediatric studies, we assessed the rate of publication of pediatric drug trials. We also examined the information that was lost when trials were not published.

## Methods

We performed a cross-sectional evaluation of pediatric trials registered in ClinicalTrials.gov. Trials were included if they examined a drug intervention in children younger than 18 years; had a randomized trial design; were registered between January 1, 2014, and June 30, 2016; and were completed or discontinued by June 30, 2018.^3^ This date was selected to allow a minimum of 2 years from trial end to publication of trial findings. The study was deemed exempt from institutional review board approval by the institutional review board at Boston Children’s Hospital because it did not involve human participants. This study followed the Strengthening the Reporting of Observational Studies in Epidemiology (STROBE) reporting guideline.

We considered a trial published if results were reported in a peer-reviewed medical journal.^4^ Publications were identified through searches in PubMed, GoogleScholar, Embase, and company websites. If no publication was found, we searched for trial reports in conference abstracts, press releases, thesis documents, trial registries, and preprint servers. We also contacted investigators to inquire about trial status and availability of trial results. Reports from unpublished studies were reviewed for findings on mortality, adverse events, and efficacy of the drug intervention. Data analysis was performed from August 28, 2020, to October 30, 2020. We used SciPy, version 1.5.0 (Python Software Foundation) to perform χ^2^ tests and Mann-Whitney tests, with statistical significance prespecified at a 2-sided *P* < .05.

## Results

Among 189 pediatric drug trials, academic institutions were the most common funding source (92 trials [48.7%]), and most (120 trials [63.5%]) included an international trial site (Table 1). Seventy-nine trials (41.8%) remained unpublished after a median follow-up period of 3.6 years (interquartile range, 3.0-4.8 years). These studies accounted for 8395 of 24 338 pediatric participants (34.5%). Publication rates at 2 and 4 years were 33.3% (63 of 189 trials) and 71.7% (109 of 152 trials), respectively.

**Table 1.** Characteristics of Published and Unpublished Trials

Characteristics	Trials, No. (%)			*P* value^a^
	Total (N = 189)	Published (n = 110)	Unpublished (n = 79)	
Status				
Completed	159 (84.1)	104 (94.5)	55 (69.6)	<.001
Discontinued	30 (15.9)	6 (5.5)	24 (30.4)	
Funding source				
Academic institutions	92 (48.7)	53 (48.2)	39 (49.4)	.93
Industry	86 (45.5)	50 (45.4)	36 (45.6)	
Health care centers	11 (5.8)	7 (6.4)	4 (5.0)	
Age group^b^				
Neonate	32 (16.9)	18 (16.4)	14 (17.7)	NA
Infant	72 (38.1)	42 (38.2)	30 (38.0)	
Child	149 (78.8)	91 (82.7)	58 (73.4)	
Adolescent	72 (38.1)	43 (39.1)	29 (36.7)	
Location				
US only	69 (36.5)	30 (27.3)	39 (49.3)	.002
US and international	32 (16.9)	25 (22.7)	7 (8.9)	
International only	88 (46.6)	55 (50.0)	33 (41.8)	
Trial phase				
Phase 1	14 (7.4)	9 (8.2)	5 (6.3)	.88
Phase 2	35 (18.5)	20 (18.2)	15 (19.0)	
Phase 3^c^	63 (33.3)	39 (35.5)	24 (30.4)	
Phase 4	48 (25.4)	27 (24.5)	21 (26.6)	
Not specified	29 (15.4)	15 (13.6)	14 (17.7)	
Masking				
Open label	28 (14.8)	12 (10.9)	16 (20.3)	.15
Single-blind	19 (10.1)	10 (9.1)	9 (11.4)	
Double-blind^d^	142 (75.1)	88 (80.0)	54 (68.4)	
Marketing status				
Marketed	160 (84.7)	97 (88.2)	63 (79.7)	.11
Premarket	29 (15.3)	13 (11.8)	16 (20.3)	
Enrollment, median (IQR), patients	68 (22-115)	79 (24-134)	55 (15-96)	.004

Abbreviations: IQR, interquartile range; NA, not applicable.

^a^ Calculated using χ^2^ tests for categorical variables and Mann-Whitney test for median values.

^b^ Neonate was defined as 0 to 1 month, infant as 1 month to 2 years, child as 2 to 12 years, and adolescent as 12 to 17 years. Categories are not mutually exclusive.

^c^ Includes 10 phase 2/3 trials.

^d^ Includes 30 trials with triple blinding and 76 trials with quadruple blinding.

Thirty trials (15.9%) were discontinued, and 6 of 30 discontinued trials (20.0%) and 104 of 159 completed trials (65.4%) were published (*P* < .001). The most frequent reasons for discontinuation were insufficient patient enrollment (10 trials [33.3%]), scientific reasons (8 trials [26.7%]), and business decisions (5 trials [16.7%]).

Trial reports were identified through online searches (n = 38) and email correspondence (n = 11) for 49 of the 79 unpublished trials (62.0%) (Table 2).^5^ Of these trials, 2 (4.1%) included deaths, 14 (28.6%) included serious adverse events, and 31 (63.3%) included nonserious adverse events. Efficacy data for the investigational drug were available in unpublished reports for 43 trials (87.8%). For the entire cohort of 79 unpublished trials, safety or efficacy data were available for a total of 44 unpublished trials (55.7%).

**Table 2.** Safety and Efficacy Data Available in Unpublished Trial Reports^a^

Drug type	Unpublished trials, No. (%)			
	All-cause mortality	Serious adverse events^b^	Any adverse events	Efficacy data
All drugs with trial reports (n = 49)	2 (4.1)	14 (28.6)	31 (63.3)	43 (87.8)
Anti-infective agents (n = 7)	NA	4 (57.1)	6 (85.7)	7 (100)
CNS agents (n = 6)	NA	1 (16.7)	4 (66.7)	6 (100)
Other drugs (n = 6)	NA	NA	1 (16.7)	5 (83.3)
Respiratory system agents (n = 5)	1 (20.0)	2 (40.0)	5 (100)	4 (80.0)
Dermatologic agents (n = 5)	NA	NA	3 (60.0)	4 (80.0)
Analgesic agents (n = 4)	NA	1 (25.0)	1 (25.0)	4 (100)
Gastrointestinal agents (n = 3)	NA	2 (66.7)	3 (100)	3 (100)
Local anesthetics (n = 3)	NA	NA	1 (33.3)	2 (66.7)
Anticoagulants and coagulants (n = 2)	1 (50.0)	1 (50.0)	1 (50.0)	2 (100)
Anti-inflammatory agents and immunosuppressants (n = 2)	NA	2 (100)	2 (100)	2 (100)
Anesthetics and sedatives (n = 2)	NA	NA	2 (100)	2 (100)
Hormones (n = 2)	NA	NA	1 (50.0)	1 (50.0)
Hypoglycemic agents (n = 1)	NA	1 (100)	1 (100)	1 (100)
Vitamins and nutrients (n = 1)	NA	NA	NA	NA

Abbreviations: CNS, central nervous system; NA, not applicable.

^a^ Trial results were identified in conference abstracts (n = 14), press releases (n = 3), thesis documents (n = 4), trial registries (n = 34), organization websites (n = 1), and preprint servers (n = 2). There were 9 trials with findings reported in more than 1 of these sources.

^b^ Serious adverse events were defined according to standard definitions provided by ClinicalTrials.gov. These include events that result in death, are life-threatening, require inpatient hospitalization, or interfere substantially with normal life functions.^5^

## Discussion

In this sample of pediatric drug trials, two-thirds remained unpublished 2 years after trial end, representing considerable loss of scientific information and inefficiency in research practices. More than half of unpublished trials generated safety and efficacy findings, which were largely inaccessible to clinicians and the scientific community.

A limitation of our study is that trial reports were available for only 62% of unpublished trials, and results may not be representative of all unpublished studies. Nonetheless, our findings point to the need for additional efforts and incentive mechanisms to ensure that pediatric trials are published in a timely fashion and that participation by children in clinical trials contributes to advances in clinical care.
